# The Leeward Islands Medical Journal

**Published:** 1892-03

**Authors:** 


*The Leeward Islands Medical Journal.* Edited by H. A. ALFORD NICHOLLS, M.D. Pp. xii., 188. London: J. & A. Churchill. 1891.

We heartily congratulate the members of the recently-formed Leeward Islands Branch of the British Medical Association upon this substantial volume, the result of good work, doubtless often done under somewhat difficult circumstances. The book, which has more the character of a volume of Transactions than of a Journal, contains many papers on subjects with which we are not very familiar here; and for these, coming from medical men with special opportunities for observation, we are grateful. The Branch has been fortunate in getting the Government to bear the expense of publishing the proceedings in book-form.

